# Rewriting Tumor Entry Rules: Microfluidic Polyplexes and Tumor-Penetrating Strategies—A Literature Review

**DOI:** 10.3390/pharmaceutics18010084

**Published:** 2026-01-09

**Authors:** Simona Ruxandra Volovat, Iolanda Georgiana Augustin, Constantin Volovat, Ingrid Vasilache, Madalina Ostafe, Diana Ioana Panaite, Alin Burlacu, Cristian Constantin Volovat

**Affiliations:** 1 Department of Medical Oncology-Radiotherapy, “Grigore T. Popa” University of Medicine and Pharmacy, 16 University Street, 700115 Iasi, Romania; simonavolovat@gmail.com (S.R.V.); ostafe.madalinaraluca@d.umfiasi.ro (M.O.);; 2 Department of Medical Oncology, University of Medicine and Pharmacy “Carol Davila”, 050474 Bucharest, Romania; 3 Department of Obstetrics-Gynecology, “Grigore T. Popa” University of Medicine and Pharmacy, 700115 Iasi, Romania; 4 Department of Thoracic Surgery, Prof. Alexandru Trestioreanu Institute of Oncology, 022328 Bucharest, Romania; 5 Department of Radiology, “Grigore T. Popa” University of Medicine and Pharmacy, 700115 Iasi, Romania

**Keywords:** cancer vaccines, non-viral gene delivery, nucleic acids, polymeric nanoparticles, cancer immunotherapy

## Abstract

Cancer immunotherapy increasingly relies on nucleic acid-based vaccines, yet achieving efficient and safe delivery remains a critical limitation. Polyplexes—electrostatic complexes of cationic polymers and nucleic acids—have emerged as versatile carriers offering greater chemical tunability and multivalent targeting capacity compared to lipid nanoparticles, with lower immunogenicity than viral vectors. This review summarizes key design principles governing polyplex performance, including polymer chemistry, architecture, and assembly route—emphasizing microfluidic fabrication for improved size control and reproducibility. Mechanistically, effective systems support stepwise delivery: tumor targeting, cellular uptake, endosomal escape (via proton-sponge, membrane fusion, or photochemical disruption), and compartment-specific cargo release. We discuss therapeutic applications spanning plasmid DNA, siRNA, miRNA, mRNA, and CRISPR-based editing, highlighting preclinical data across multiple tumor types and early clinical evidence of on-target knockdown in human cancers. Particular attention is given to physiological barriers and engineering strategies—including size-switching systems, charge-reversal polymers, and tumor-penetrating peptides—that improve intratumoral distribution. However, significant challenges persist, including cationic toxicity, protein corona formation, manufacturing variability, and limited clinical responses to date. Current evidence supports polyplexes as a modular platform complementary to lipid nanoparticles in selected oncology indications, though realizing this potential requires continued optimization alongside rigorous translational development.

## 1. Introduction

Cancer care has witnessed striking advances over the last century, including the eradication of several lethal infectious diseases and the advent of effective therapies for many others; nonetheless, cancer remains an unresolved clinical challenge. In 2022, there were an estimated ~20 million new cancer cases and 9.7 million deaths worldwide, with 53.5 million individuals alive within five years of a cancer diagnosis. Lifetime risk remains high—approximately one in five people will develop cancer—and mortality remains substantial, at roughly 1 in 9 men and 1 in 12 women [1]. Despite scientific progress, no single curative strategy exists for the spectrum of malignancies. Worryingly, global incidence and mortality continue to climb, with projections indicating ~10 million cancer deaths annually and potentially up to 15.4 million by 2030. These trajectories intensify pressure on governments and health systems and, in parallel, on the scientific community to accelerate the development of innovative anticancer modalities with superior therapeutic efficacy [2].

Conventional treatments—chemotherapy, radiotherapy, and surgery—remain the global standard of care and can yield favorable outcomes depending on histology and stage. Yet each modality carries intrinsic limitations: cytotoxic agents damage healthy tissues and drive systemic adverse effects; radiotherapy and surgery may fail to eradicate micrometastatic disease or reach all malignant foci, leaving room for relapse and dissemination. Consequently, there is sustained demand for cost-effective, scalable interventions that are both broadly accessible and mechanistically precise. In this context, cancer vaccine–based immunotherapy has garnered increasing attention, aiming to mobilize and educate the host immune system to recognize and eliminate malignant cells. By enabling antigen-specific cytotoxicity with reduced off-target injury, limiting metastatic spread, and establishing durable immune memory, vaccine strategies offer conceptual advantages over purely cytotoxic approaches [3]. However, the clinical success of nucleic acid–based cancer vaccines—encoding tumor antigens, immunostimulatory molecules, or gene-editing components—depends critically on delivery systems capable of protecting cargo from degradation, achieving tumor-specific accumulation, and ensuring intracellular release at the site of action [4].

Several platform technologies compete for this role. Viral vectors offer high transfection efficiency but carry risks of immunogenicity, insertional mutagenesis, and manufacturing complexity [5]. Lipid nanoparticles (LNPs) have achieved clinical validation through COVID-19 mRNA vaccines, yet face limitations including preferential hepatic accumulation, inflammatory responses, and anti-PEG immunity upon repeated dosing [6]. Exosomes and hybrid systems offer biocompatibility but present standardization and scalability challenges [7].

Polyplexes—electrostatic complexes of cationic polymers and nucleic acids—represent a distinct alternative with potential advantages for cancer vaccine delivery. Compared to viral vectors, they exhibit lower immunogenicity and no integration risk. Relative to LNPs, polyplexes offer, in principle, greater chemical diversity for backbone modification, higher ligand density enabling multivalent receptor engagement, and superior compatibility with stimuli-responsive design. However, these theoretical advantages have not yet been validated in comparative clinical trials, and LNPs currently represent the only clinically approved platform for systemic nucleic acid delivery in oncology [8]. Critically, polymer chemistry permits engineering of systems that respond to tumor microenvironment cues—including acidic pH (6.5–6.8), elevated glutathione (redox), hypoxia, or overexpressed enzymes (e.g., matrix metalloproteinases)—enabling site-specific cargo release [9].

Two aspects of polyplex technology are particularly relevant to overcoming delivery barriers in solid tumors. First, microfluidic assembly enables precise control over mixing kinetics, yielding polyplexes with narrower size distributions (polydispersity index < 0.1) and enhanced batch reproducibility compared to conventional bulk mixing—attributes critical for clinical translation and regulatory approval [10]. Second, tumor-penetrating strategies—including size-switching nanoparticles, charge-reversal systems, and peptides engaging the neuropilin-1/CendR pathway (e.g., iRGD)—can be integrated into polyplex design to address the dense stroma, elevated interstitial pressure, and heterogeneous vascularization that limit intratumoral distribution [11].

The present review provides a critical analysis of polymer-based polyplexes as nucleic acid delivery platforms for cancer therapy. We examine structure–function relationships governing polyplex performance, compare microfluidic versus conventional assembly methods, analyze mechanisms of cellular entry and endosomal escape, and evaluate therapeutic strategies across nucleic acid modalities (pDNA, siRNA, miRNA, mRNA, CRISPR). Particular attention is given to physiological barriers limiting clinical translation and engineering strategies to overcome them. Rather than cataloguing advances uncritically, we aim to identify where polyplexes offer genuine advantages over competing platforms and where significant challenges remain unresolved.

## 2. Structure of Polyplexes

Polyplexes are nanostructured complexes formed through electrostatic association between negatively charged nucleic acids and positively charged (cationic) polymers. This self-assembly process is thermodynamically favorable, driven primarily by counterion release (entropic gain) and Coulombic attraction between the oppositely charged species [12]. Unlike lipid-based systems requiring precise lipid:cargo ratios and complex formulation protocols, polyplexes form spontaneously upon mixing under appropriate conditions, simplifying initial preparation. The resulting colloidal stability depends on the balance between attractive forces (electrostatic, van der Waals, hydrophobic interactions) and repulsive forces (steric repulsion from hydrophilic shells, electrostatic repulsion from surface charge), with stability under physiological ionic strength (150 mM NaCl) representing a key formulation challenge [13].

**Figure 1:**
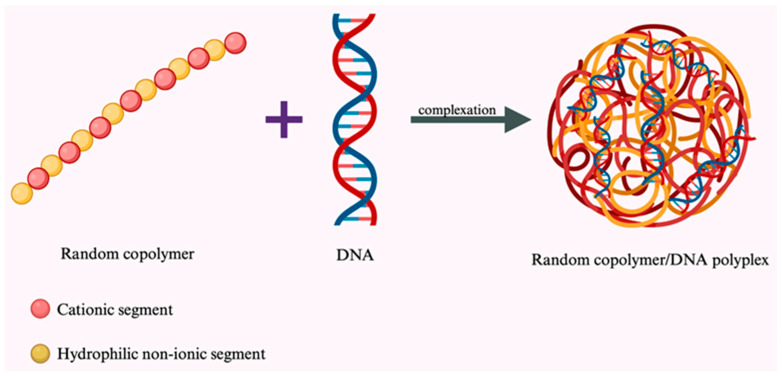
Schematic illustration of the formation of polymer/DNA polyplexes. A random copolymer composed of cationic segments and hydrophilic non-ionic segments interacts electrostatically with negatively charged DNA. Upon complexation, the polymer chains and DNA condense into a compact, nanoscale random copolymer/DNA polyplex, in which multiple DNA strands are entangled and shielded by the mixed cationic–hydrophilic polymer segments.

During formation, polyplexes adopt various morphologies including globular, spherical, toroidal, and rod-like structures [14]. This morphological diversity arises from the interplay between polymer architecture, nucleic acid topology, and assembly kinetics (Figure 1). A comprehensive analysis of polyplex structure revealed that the extent of condensation depends primarily on the following factors: (i) the physicochemical properties of the cationic polymer, including molecular weight, charge density, and pKa of ionizable groups; (ii) polymer architecture—linear versus branched or dendritic; (iii) the nitrogen-to-phosphate (N/P) ratio, which determines net surface charge; and (iv) the preparation method employed [14]. Researchers have observed that polymers with substantial branching produce polyplexes with reduced particle size and enhanced transfection efficiency, though often at the cost of increased cytotoxicity [15]. The general mechanism of polyplex formation through electrostatic complexation be-tween cationic polymers and nucleic acids is illustrated in Figure 1.

### 2.1. Cargo-Dependent Structural Differences

Polyplex structure varies substantially with nucleic acid type, with direct implications for delivery and tumor penetration:

*Plasmid DNA* (*pDNA*) is circular, double-stranded, and relatively large (typically 5–10 kb, ~3–6 MDa). Upon complexation with cationic polymers, pDNA condenses into compact toroidal or spheroidal structures typically ranging from 100 to 300 nm. These larger complexes provide effective cargo protection but face significant challenges in penetrating dense tumor stroma [16].

*Messenger RNA* (*mRNA*) is single-stranded with extensive secondary structure (hairpins, loops) that influences condensation behavior. mRNA polyplexes tend to be somewhat less compact than pDNA complexes and exhibit greater sensitivity to RNase degradation, necessitating rapid and complete complexation. Modified nucleotides (e.g., pseudouridine, N1-methylpseudouridine) further alter condensation properties [17].

*Small interfering RNA* (*siRNA*) consists of short (21–23 bp), rigid double-stranded segments. Due to their low molecular weight (~14 kDa) and limited charge density, siRNAs often require higher N/P ratios or additional stabilization strategies (lipid modification, crosslinking) for efficient complexation. However, the resulting polyplexes are typically smaller (20–80 nm), which confers advantages for tumor penetration [16].

*MicroRNA* (*miRNA*) mimics and inhibitors behave similarly to siRNA in complexation, though chemical modifications for stability may alter binding characteristics [16].

### 2.2. Structural Determinants of Tumor Penetration

The structural characteristics of polyplexes directly influence their ability to navigate the tumor microenvironment—a central consideration for therapeutic efficacy.

*Size* is a critical determinant. Optimal thresholds vary with tumor type and matrix composition. In several preclinical models, particles below ~100 nm penetrate tumor interstitium more effectively, while those exceeding 200 nm remain largely confined to perivascular regions [8]. Comparative studies in ovarian cancer spheroid models demonstrate that smaller siRNA polyplexes (~25 nm) penetrate significantly deeper than larger pDNA polyplexes (~160 nm), which remain at the spheroid periphery; however, these thresholds are model-dependent and may differ in tumors with varying stromal density [18].

*Surface charge* influences both extravasation and interstitial transport. While cationic surfaces facilitate cellular uptake, they also promote nonspecific binding to anionic extracellular matrix components (collagen, hyaluronan, proteoglycans), potentially trapping particles near the vasculature. Charge-shielding strategies (PEGylation, zwitterionic coatings) can improve matrix penetration but may compromise uptake efficiency—a tradeoff requiring careful optimization [19].

*Particle rigidity* also affects penetration. Softer, more deformable particles may navigate tortuous interstitial paths more effectively than rigid counterparts, though systematic studies in polyplex systems remain limited [20].

In summary, polyplex structure is not merely a physicochemical parameter but a critical determinant of biological performance. Rational design must consider cargo type, target tissue characteristics, and the specific barriers to be overcome—with smaller, appropriately charged particles generally favoring deep tumor penetration at the potential cost of reduced cargo capacity or stability.

## 3. Types of Polymers in Polyplexes

**Figure 2:**
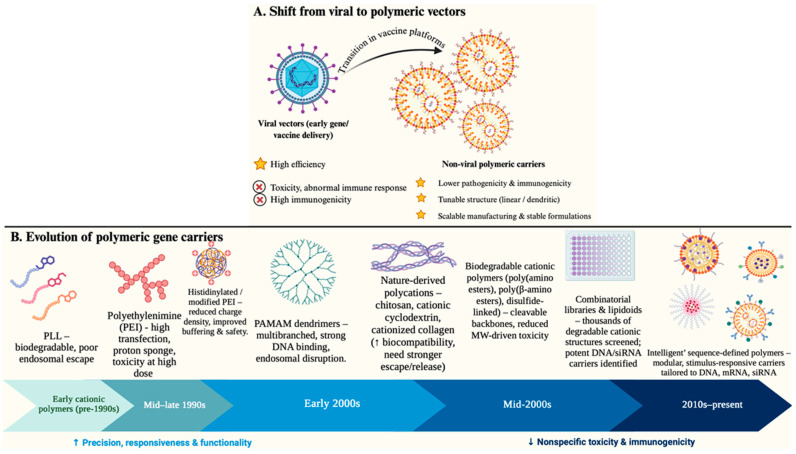
Evolution of non-viral polymeric gene delivery systems. (**A**) Conceptual shift from viral to non-viral vectors. Viral vectors offer high transfection efficiency but are limited by toxicity and strong immune responses. Advances in macromolecular chemistry and understanding of delivery barriers have enabled non-viral polymeric carriers with lower pathogenicity, tunable linear or dendritic structures, improved biocompatibility, and scalable, stable formulations. (**B**) Timeline of major polymer classes for nucleic acid delivery. Early poly(L-lysine) (PLL) was biodegradable but inefficient in endosomal escape and immunogenic. Polyethylenimine (PEI) marked a breakthrough with high transfection and proton-sponge activity but dose-dependent toxicity. Subsequent developments focused on charge-reduced and buffered PEI derivatives, dendrimers (PAMAM), and nature-derived polymers (e.g., chitosan, cationic cyclodextrins, cationized collagens) to improve safety. Biodegradable cationic polymers with cleavable linkages and high-throughput libraries of degradable polymers and lipidoids further enhanced performance. Current “intelligent,” sequence-defined polymers integrate cationic, shielding, biodegradable, and targeting domains for stimulus-responsive delivery of DNA, mRNA, or siRNA.

Significant advances in macromolecular chemistry, together with a clearer understanding of extra- and intracellular delivery bottlenecks, have enabled several foundational innovations in nucleic-acid transport [21]. Motivated by toxicity and aberrant immune responses observed with early viral vectors, contemporary vaccine platforms increasingly favor non-viral polymeric carriers. These materials—available as linear chains or branched/dendritic architectures—combine low pathogenicity and immunogenicity with tunable biocompatibility, mechanical robustness, manufacturability, and formulation stability, all of which are essential for effective gene delivery [22]. An unifying design principle emerging from decades of research is that polyplexes must be dynamic: they should tightly condense nucleic acids extracellularly, enable endosomal escape upon acidification, and then support intracellular release in the correct compartment (cytosol for mRNA/siRNA; nucleus for pDNA), with task requirements differing between cargo types [23]. Figure 2 illustrates the conceptual evolution from viral to non-viral vectors (A) and the historical timeline of major polymer classes developed for nucleic acid delivery (B).

### 3.1. Polyethylenimine (PEI): Historical Benchmark and Contemporary Limitations

The adoption of polyethylenimine (PEI) in 1995 represented a pivotal moment in non-viral gene delivery: its chemical simplicity, extracellular condensation strength, and reliable in vitro/in vivo transfection established PEI as a widely used transfection agent. The “proton sponge” hypothesis—whereby PEI’s high buffering capacity (pKa ~ 6–7) drives osmotic endosomal rupture—provided a mechanistic framework that guided subsequent polymer design [24]. However, PEI’s clinical translation has been fundamentally limited by dose-dependent toxicity. Branched PEI (25 kDa) induces a biphasic cytotoxic response: an early phase (<30 min) characterized by plasma membrane disruption and LDH release, followed by a delayed phase (~24 h) involving mitochondrial depolarization, cytochrome-c release, and caspase-3 activation [25]. Toxicity scales with molecular weight (25 kDa >> 2 kDa) and branching architecture (branched > linear), creating a fundamental efficiency-toxicity tradeoff [26].

Beyond acute toxicity, regulatory and manufacturing challenges have impeded PEI’s clinical development. Structural heterogeneity inherent to polymerization processes results in batch-to-batch variability that complicates quality control and regulatory approval [27]. Chronic accumulation in liver and kidneys—due to PEI’s non-biodegradability—raises long-term safety concerns inadequately addressed by acute toxicity studies. Consequently, while PEI remains valuable as a laboratory benchmark, its direct clinical relevance for systemic oncology applications is limited, and contemporary development has shifted toward safer alternatives [28].

Modification strategies have partially addressed these limitations. Histidinylated linear PEI improves buffering while reducing toxicity. PEGylation extends circulation but creates the “PEG dilemma”—reduced cellular uptake and potential anti-PEG immunity. Crosslinking with biodegradable disulfide bonds enables intracellular degradation into less toxic fragments [29].

### 3.2. Poly(L-lysine) (PLL) and Polypeptide Systems

Early cationic polypeptides such as poly(L-lysine) (PLL) illustrated both promise and constraint: intrinsically biodegradable and biocompatible through enzymatic hydrolysis, yet limited by inefficient endosomal escape rooted in poor buffering capacity at endosomal pH. Under physiological conditions, lysine residues are fully protonated (pKa ~ 10.5), leaving no buffering capacity in the endosomal pH range (5.5–6.5) to drive osmotic rupture [14,30].

Systematic PLL modification has generated derivatives with improved performance: PEGylation reduces opsonization; histidine incorporation provides buffering capacity; incorporation of hydrophobic domains enhances membrane interaction [31]. Contemporary polypeptide systems exploit recombinant or solid-phase synthesis to create sequence-defined structures with precisely positioned functional domains—a significant advance over polydisperse traditional polymers [32].

### 3.3. Poly(amidoamine) Dendrimers (PAMAM)

Dendritic poly(amidoamine) (PAMAM) demonstrates how multibranch topology and precisely controlled surface charge facilitate complexation and promote endosomal interactions favoring cargo release [33]. Unlike linear polymers, dendrimers are monodisperse with generation-dependent size and surface amine density, enabling structure–activity relationships impossible with heterogeneous polymers. However, higher-generation dendrimers (G5–G7) exhibit significant toxicity correlating with surface amine density. Partial acetylation or hydroxylation of surface amines mitigates toxicity while maintaining complexation capacity. Manufacturing costs and synthetic complexity increase exponentially with generation number, presenting scalability challenges for clinical translation [34].

### 3.4. Biodegradable Cationic Polymers

*Poly(β-amino ester)s (PBAEs)* represent a major advance in biodegradable carriers. Synthesized through the Michael addition of amines to diacrylates, PBAEs hydrolyze at physiological pH into non-toxic small molecules, dramatically reducing accumulation-related toxicity [35]. Combinatorial library screening—wherein thousands of structurally diverse polymers are synthesized by systematically varying amine and diacrylate monomers—has identified structures approaching viral transfection efficiency with favorable safety profiles [36]. However, batch-to-batch variability and hydrolytic instability during storage remain translational challenges requiring controlled manufacturing environments [37].

*Disulfide-crosslinked polymers* exploit the reducing intracellular environment (2–10 mM glutathione versus < 10 μM extracellular) to trigger polymer degradation and cargo release selectively within cells. This “bioreducible” strategy maintains extracellular stability while ensuring intracellular unpacking [38].

### 3.5. Nature-Derived Polymers

To improve biocompatibility and biodegradability, nature-derived systems have been extensively explored. *Chitosan derivatives* (from chitin deacetylation) offer biocompatibility, biodegradability, and mucoadhesive properties. However, chitosan’s pKa (~6.5) results in poor solubility and reduced protonation at physiological pH [39]. Trimethylation or quaternization maintains permanent positive charge, improving complexation efficiency [40]. *Cationic cyclodextrin conjugates* provide hydrophobic cavities for drug co-loading alongside nucleic acid complexation [41]. *Atelocollagen* has demonstrated efficacy for siRNA delivery in bone metastasis models [42].

Naturally occurring cationic proteins (e.g., protamine, histones) and polysaccharides (e.g., dextran, pullulan) carry charge densities conducive to complexation while retaining favorable safety profiles. These materials often show enhanced transfection relative to purely synthetic comparators in vaccine applications [43].

### 3.6. Sequence-Defined Polymers

A significant recent advance is the development of sequence-defined polymers—macromolecules with precisely specified monomer sequences analogous to peptides or oligonucleotides, synthesized through solid-phase or iterative solution-phase methods [44]. Unlike conventional polymers with statistical monomer distributions, sequence-defined systems enable the following: (i) exact positioning of cationic, hydrophobic, and targeting domains along the polymer backbone; (ii) reproducible structure–activity relationships impossible with polydisperse materials; and (iii) the incorporation of specific functional sequences (nuclear localization signals, endosomolytic peptides, targeting ligands) at defined positions [45]. For example, sequence-defined oligo(ethanamino)amides enable systematic optimization of charge distribution, hydrophobicity, and topology for specific cargos (pDNA versus siRNA versus mRNA) [46]. The primary limitation is synthetic complexity: current methods require labor-intensive iterative coupling, restricting scale-up and increasing costs relative to conventional polymerization [47]. A comparative overview of the key polymer classes discussed above, including their physicochemical properties and principal limitations, is provided in Table 1.

## 4. Formation of Polyplexes

Polyplexes form by electrostatic complexation between cationic polymers and nucleic acids, typically initiated by protonated amines on the carrier. The central rationale is straightforward: naked nucleic acids are rapidly degraded by nucleases in vivo, whereas nucleic acids complexed with polycations are sterically and electrostatically shielded, gaining stability while avoiding the immunogenicity typical of viral vectors. In practice, this enables robust, non-viral protection and delivery of anionic genetic cargo [48].

### 4.1. Principles Governing Complexation

Polyplex formation relies fundamentally on electrostatic interactions between protonated amines on the polymer and phosphate groups on the nucleic acid backbone. However, the final complex stability and properties emerge from a hierarchy of interacting forces [12]:

*Primary electrostatic interactions* provide the driving force for initial association. The nitrogen-to-phosphate (N/P) ratio—the molar ratio of protonatable amines to nucleic acid phosphates—is the most critical formulation parameter. N/P ratios of 3–10 are typical; values below this threshold result in incomplete condensation and unstable complexes, while excessive ratios increase free polymer concentration and associated toxicity [19].

*Counterion release* provides a significant entropic contribution to binding thermodynamics. As cationic polymer associates with anionic nucleic acid, condensed counterions (Na^+^, Cl^−^) are displaced into bulk solution, increasing system entropy and favoring complex formation [13].

*Hydrophobic interactions* contribute to complex stability when polymers contain aliphatic or aromatic domains. These secondary interactions can enhance condensation strength and influence intracellular unpacking kinetics [26].

*Hydrogen bonding* between polymer functional groups and nucleic acid bases or backbone provides additional stabilization in some systems, particularly for peptide-based carriers [49].

The relative dominance of these forces depends on polymer chemistry and environmental conditions. Ionic strength is particularly important: physiological salt concentrations (150 mM NaCl) partially screen electrostatic interactions, weakening complexes optimized at low ionic strength and necessitating formulation under conditions mimicking the biological environment [8].

### 4.2. Polymer-Specific Determinants

Beyond the fundamental electrostatic interactions, polymer molecular weight, charge density and distribution, architecture, chemical functionality, and chain mechanics collectively govern polyplex binding strength, particle size, colloidal stability, cellular uptake, endosomal escape, and ultimately transfection efficiency. Understanding these interrelated parameters is essential for rational carrier design, as optimal formulations must balance strong extracellular binding against efficient intracellular release—a fundamental trade-off underlying all polyplex systems [12].

Molecular weight represents perhaps the most accessible optimization parameter, yet its effects are nuanced and non-linear. Low molecular weight polymers typically exhibit weak nucleic acid binding and incomplete condensation, yielding loose, unstable polyplexes with poor cargo protection; however, they generally show lower cytotoxicity and more facile intracellular release [50]. At the opposite extreme, high molecular weight polymers increase binding affinity and particle stability but tend to elevate cytotoxicity and impede payload release, such that very high molecular weight frequently proves detrimental to net transfection despite strong condensation. The optimal compromise often lies at intermediate molecular weights. Recent work with highly branched-linear poly(β-amino ester)s (H-LPAEs) illustrates this principle: polymers at approximately 11.5 kDa produced small, positively charged polyplexes exhibiting high cellular uptake and superior transfection efficiency in both adherent and suspension cell lines while maintaining favorable biocompatibility [51]. Importantly, absolute polymer mass and solution conditions during complexation further modulate these molecular weight effects, and no universal thresholds exist—optimal values depend on polymer chemistry, cargo type, and target cell population.

Charge density and its spatial distribution along the polymer backbone exert equally profound influences on polyplex behavior. Higher overall cationic charge density strengthens nucleic acid condensation and nuclease protection but simultaneously increases protein adsorption and cytotoxicity. Charge density also determines buffering capacity, which directly impacts endosomal escape efficiency [52]. Beyond total charge, its distribution matters considerably: architectures presenting distributed rather than clustered charges—such as graft or brush configurations—can complex DNA efficiently while reducing cytotoxicity and improving nucleic acid release compared with densely packed linear charge arrays [53]. Furthermore, optimal charge requirements differ substantially between cargo types. The low total charge of siRNA typically necessitates higher N/P ratios for stable complexation, whereas larger pDNA molecules tolerate lower ratios; polymer composition must therefore be tailored to the specific nucleic acid and its required release profile [54].

Polymer architecture—whether linear, branched, dendritic, star, or bottlebrush—fundamentally reshapes how carriers wrap, shield, and present charges to nucleic acids and biological interfaces. Comparative studies consistently demonstrate that non-linear architectures often outperform linear analogues by enhancing complexation efficiency or reducing toxicity. Linear polymers achieve straightforward condensation with variable particle sizes depending on molecular weight and charge density, and can yield high transfection but frequently exhibit elevated cytotoxicity at equivalent charge levels. Branched and hyperbranched polymers present multivalent charges that improve compaction and reduce particle size, often enhancing uptake and endosomal escape, though molecular weight and branching degree must be carefully tuned to avoid toxicity. Dendritic structures such as PAMAM achieve strong multivalent binding and compact complexes with high binding constants, particularly at higher generations, but risk cytotoxicity and hindered release unless surface-modified. Star and nano-star architectures present multiple interaction points through their arms, with micellar stars sometimes outperforming covalent analogues depending on arm length and grafting density. Perhaps most promisingly, bottlebrush and graft copolymers present distributed charges through dense side-chains while providing steric shielding, producing either elongated or compact structures that demonstrate improved complexation, reduced cytotoxicity, and transfection efficiency comparable to PEI in optimized formulations. Architecture thus controls local charge presentation, steric protection from serum proteins, and polymer-DNA packing geometry, making it a critical design variable for balancing stability against release [53].

Chemical composition and chain mechanics further modulate these structure–function relationships. Primary, secondary, and tertiary amines provide both electrostatic binding and pH-dependent buffering capacity that promotes endosomal escape through the proton sponge mechanism. Modulating amine type and local chemical environment alters buffering capacity and membrane interaction profiles; tertiary amine-rich backbones often show improved buffering with reduced direct membrane disruption compared with primary amine-dominated polymers [50]. Strategic chemical modifications can dramatically improve performance: incorporation of oxazoline and ethyl-oxazoline segments into PEI-like copolymers has produced third-generation materials achieving high transfection with low cytotoxicity, attributed to altered protein interactions and improved endosomal release relative to unmodified PEI [55]. Hydrophobic-hydrophilic balance requires careful optimization, as moderate hydrophobic segments enhance membrane interactions and endosomal escape but excessive hydrophobicity increases aggregation and serum protein binding, compromising circulation stability [54]. Chain flexibility also matters: flexible backbones permit tighter nucleic acid wrapping and more compact polyplexes, while rigid or bulky side chains produce less compact or elongated morphologies and alter release kinetics. For naturally derived polymers such as chitosan, performance depends on molecular weight, degree of deacetylation, and specific functionalization—parameters that collectively modulate binding affinity, targeting capability, and stimulus-responsive release [54].

Integrating these determinants reveals the central trade-off governing polyplex design: stronger binding through high molecular weight, elevated charge density, multivalent architecture, or hydrophobic patches increases stability and nuclease protection while tending to reduce particle size, but overly strong binding, high cytotoxicity, or excessive steric hindrance impedes intracellular release and ultimately reduces transfection efficiency. The design objective is therefore not maximal binding but rather sufficient condensation and serum stability paired with efficient intracellular unpacking. Small, compact polyplexes—typically produced by intermediate molecular weight polymers with optimized branching—favor cellular uptake and circulation stability while permitting adequate cargo release. Architectures that present charges while sterically shielding the particle core, such as bottlebrush and graft copolymers, can achieve PEI-level transfection while substantially lowering cytotoxicity and reducing problematic serum protein interactions [53].

From this body of work, several practical design principles emerge. First, intermediate molecular weight or degradable high molecular weight polymers should be favored to achieve tight yet releasable complexes. Second, charge density and distribution should be tuned to balance effective binding against reduced serum interactions and cytotoxicity, with cargo-specific optimization essential. Third, non-linear architectures—particularly branched, graft, and bottlebrush configurations—offer advantages in enhancing complexation while lowering toxicity or enabling triggered unpacking. It must be acknowledged, however, that specific quantitative thresholds—exact molecular weight windows or charge density values—that universally maximize transfection across cell types and applications are not consistently established in the literature. Optimal parameters remain dependent on the specific polymer chemistry, nucleic acid cargo, formulation conditions, and biological context, precluding universal numeric recommendations [46,53].

### 4.3. Cargo-Specific Complexation Requirements

Effective polyplex design must be matched to the physicochemical properties of the nucleic acid cargo. Compact, high-charge condensation and prolonged stability are prioritized for plasmid DNA delivery to the nucleus; protection from degradation combined with facile release and robust endosomal escape are essential for siRNA; and preservation of structural integrity with efficient cytoplasmic release governs mRNA delivery. Polymer architecture, molecular weight, and charge density represent the principal tuning parameters for achieving these cargo-specific objectives [12].

#### 4.3.1. Plasmid DNA Requirements

Plasmid DNA presents unique delivery challenges arising from its high molecular weight, flexible polyanionic character, and nuclear destination. Successful pDNA polyplexes must achieve strong multivalent condensation to collapse these large molecules into nanoscale particles while maintaining prolonged stability through extracellular transit and endosomal escape, yet permit controlled unpacking at or within the nucleus. The size and flexibility of plasmids—typically 5–10 kilobases encoding 3–6 megadaltons—demand multivalent polycations capable of cooperative binding along the extended DNA contour. Polymer molecular weight and topology strongly govern binding strength and the resulting polyplex size and surface charge [46,50]. Recent work with intermediate molecular weight branched-linear poly(β-amino ester)s demonstrates this principle: polymers at approximately 11.5 kDa produced small, well-compacted DNA polyplexes achieving exceptionally high transfection in suspension cells, confirming that molecular weight and topology tuning is critical for pDNA delivery [46].

Effective plasmid complexation requires sufficient excess cationic charge to neutralize and overcompensate phosphate groups, driving condensation and ensuring colloidal stability. Experimental performance often depends on absolute polycation amount as well as nominal N/P ratio, with a practical minimum polymer dose necessary to ensure complete charge compensation regardless of calculated stoichiometry [56]. Formulations therefore target net-positive polyplexes to promote cellular binding while carefully tuning polymer architecture and dose to avoid excessive cytotoxicity [56].

Tight compaction serves multiple functions: it reduces plasmid persistence length, protects DNA from shear forces during processing and administration, and shields against nuclease attack. Polymers providing multivalent, cooperative binding—including PAMAM dendrimers, branched PEI, and bottlebrush architectures—produce compact particles and can modulate release kinetics through grafting density or incorporation of degradable linkages [53]. Stability must be sufficient to survive extracellular nucleases and endosomal transit but permit unpacking near or inside the nucleus. Design strategies addressing this balance include buffering capacity for endosomal escape combined with stimuli-cleavable linkers—pH-sensitive or redox-responsive bonds—that trigger DNA release at appropriate subcellular locations [50]. Bottlebrush copolymers exemplify this approach, demonstrating improved pDNA complexation and nucleic acid release compared with linear polymers while simultaneously reducing cytotoxicity [53].

#### 4.3.2. siRNA Requirements

Small interfering RNA presents fundamentally different complexation challenges. As short, rigid double-stranded molecules of only 21–23 base pairs, siRNAs require protection from ubiquitous serum nucleases and efficient cytosolic release to permit incorporation into the RNA-induced silencing complex (RISC). Polyplexes for siRNA therefore prioritize shielding, rapid cellular uptake, and facile intracellular unpacking rather than the prolonged stability required for pDNA. The inherently short length of siRNA yields fewer phosphate binding sites per molecule, reducing multivalency and favoring weaker polymer binding per particle compared with plasmid complexes. Formulations consequently often load multiple siRNA molecules per polycation aggregate or employ architectures that scaffold several oligonucleotides to achieve stable particle formation [57,58]. Because each siRNA contributes limited negative charge, polymers with high local charge density or multivalent presentation—dendrimers, densely grafted brushes, or multiblock copolymers—are typically required to achieve stable condensation [53,55].

Protection from degradation relies on shielding by the polycation combined with steric stabilization through PEGylation or grafted side chains, maintaining siRNA integrity until cytoplasmic delivery [50]. Rapid endosomal escape is particularly critical for siRNA because the cargo must reach the cytosol intact; polymers incorporating buffering capacity for proton sponge-mediated escape, membrane-active segments, or pH-responsive motifs that promote endosomal disruption during acidification are commonly employed [59].

Optimal siRNA carriers thus feature high local charge density, moderate molecular weight to limit toxicity, architectures presenting multiple cationic groups, and stimuli-labile linkages permitting cytosolic release [55]. Importantly, polymer compositions optimized for pDNA are not automatically suitable for siRNA; studies consistently demonstrate that polymer chemistry must be re-tuned for short RNAs to appropriately balance protection against release requirements [60].

#### 4.3.3. mRNA Requirements

Messenger RNA occupies an intermediate position in terms of molecular size but presents perhaps the most stringent requirements for structural preservation. mRNA molecules range from hundreds to several thousand nucleotides, creating high molecular weight and extended conformations that demand carriers capable of packaging long chains into colloidally stable particles without extensive shear or denaturation. Unlike DNA, which is relatively robust, mRNA function depends critically on preservation of secondary structure, 5′-cap accessibility, and poly(A) tail integrity—damage to any of these features during complexation abolishes translational competence. Polyplexes for mRNA must therefore employ gentle compaction strategies. Mild complexation chemistries relying on non-covalent electrostatic assembly are typically used to avoid damaging the cargo [50,55]. Beyond protection, polymers must release mRNA in the cytosol fully intact without occluding ribosome access; stimuli-responsive linkages or low-affinity binding motifs that permit facile dissociation under cytosolic conditions therefore improve translation efficiency [55].

#### 4.3.4. Other Nucleic Acid Cargos

MicroRNA mimics, antisense oligonucleotides, and aptamers occupy an intermediate spectrum of size and structural motifs, requiring polymer selection matched to each cargo’s specific length, rigidity, and intracellular target. MicroRNA and antisense oligonucleotides, as short single- or double-stranded species, require strong local charge presentation for complexation but also need facile cytosolic release; moderate-affinity polycations incorporating endosomal escape motifs and steric shielding often perform optimally [50,55]. Aptamers present additional challenges: these structured single-stranded oligonucleotides with defined tertiary folding require particularly gentle, non-denaturing complexation to preserve binding activity, favoring lower-affinity, reversible electrostatic interactions or protective encapsulation strategies [50].

#### 4.3.5. Integration: Matching Cargo Properties to Design Decisions

Cargo properties map to design decisions in predictable ways, and successful systems adjust polymer chemistry, architecture, and formulation parameters to match these attributes. For plasmid DNA, multivalent moderate-to-high molecular weight polycations or architectures enabling strong cooperative binding—PEI, PAMAM, PBAE, bottlebrush configurations—are required to condense long flexible chains into compact, dense nanoparticles typically ranging from tens to low hundreds of nanometers. Sufficient cation excess must overcompensate phosphate charge, with absolute polycation dose often more important than nominal N/P ratio in practice. Stability must be maintained until nuclear delivery, with stimuli-triggered unpacking or buffering-mediated endosomal escape enabling nuclear access [50].

For siRNA, high local charge density architectures with shielding and endosomal escape motifs produce small, highly uniform particles, often requiring multimerization of siRNA molecules per particle for stability. Moderate N/P ratios sufficient to bind multiple siRNA copies per carrier should be tuned to allow rapid cytosolic release after endosomal escape, with fast unpacking and preservation of duplex integrity supporting efficient RISC loading [61,62].

For mRNA, gentle protective polymers that preserve secondary structure and permit full cytosolic dissociation—degradable PBAEs, bottlebrush copolymers, PEG-stabilized systems—produce larger polyplexes or structured complexes maintaining mRNA integrity. Formulations must provide sufficient polymer to protect long chains while avoiding tight binding that blocks ribosomal access; dose and architecture tuning is critical. Robust endosomal escape followed by complete unpacking exposes the cap and poly(A) tail for translation [63,64].

Efficiency emerges from integrated performance across extracellular stability, cellular uptake, endosomal escape, and timely unpacking. Polymer molecular weight and architecture tuning has produced very high efficiencies in model systems—H-LPAEs with intermediate molecular weight achieved exceptional DNA transfection, while bottlebrush copolymers improved pDNA complexation and lowered cytotoxicity [53,64]. These examples underscore that optimal polyplex design is not universal but must be deliberately matched to the specific nucleic acid cargo, its intracellular destination, and the desired therapeutic outcome.

### 4.4. Assembly Methods: Impact on Polyplex Properties

The method by which polymer and nucleic acid solutions are combined exerts substantial influence on the resulting polyplex properties, reproducibility, and ultimately clinical translatability. While the fundamental electrostatic interactions driving complexation remain constant regardless of assembly approach, the kinetics of mixing and local concentration gradients during particle formation determine size distribution, morphology, and batch consistency. Conventional bulk mixing—whether by pipetting, vortexing, or simple addition of one solution to another—remains the predominant laboratory method due to operational simplicity. However, this approach inherently produces heterogeneous local concentrations during the mixing process, as polymer-rich and nucleic acid-rich microdomains transiently coexist before homogenization. The consequence is polydisperse particle populations with polydispersity indices (PDI) typically ranging from 0.2 to 0.4, accompanied by significant batch-to-batch variability that complicates quality control and regulatory evaluation [65]. These limitations become increasingly problematic as formulations advance toward clinical development, where reproducibility and defined critical quality attributes are essential for regulatory approval.

Microfluidic assembly addresses these limitations through precise engineering control over mixing kinetics. In microfluidic devices, polymer and nucleic acid streams flow through defined channel geometries—typically Y-junctions, T-junctions, or more sophisticated herringbone or staggered structures—and combine under laminar flow conditions where mixing occurs primarily through diffusion across well-defined interfaces [66]. This controlled environment produces uniform local concentrations throughout the mixing zone, yielding substantially narrower size distributions with PDI values consistently below 0.1 in optimized systems [67].

The advantages of microfluidic fabrication extend beyond particle uniformity. Flow rate ratios between polymer and nucleic acid streams provide a tunable parameter for controlling final particle size independent of formulation composition, enabling systematic optimization [12]. Batch-to-batch reproducibility improves dramatically when mixing conditions are precisely defined by device geometry and pump settings rather than operator technique. Scalability, initially perceived as a limitation of microfluidic approaches, has been addressed through parallelization strategies employing multiple channels operating simultaneously, enabling production volumes compatible with preclinical and early clinical studies. Furthermore, continuous-flow microfluidic production is inherently compatible with in-line analytical monitoring and quality control, facilitating process analytical technology (PAT) implementation required for GMP manufacturing [68,69].

Despite these manufacturing advantages, direct evidence that microfluidic preparation translates to improved therapeutic outcomes in cancer applications is currently lacking. Comparative studies have demonstrated more consistent biodistribution profiles for microfluidic-prepared particles [70], but head-to-head efficacy comparisons with bulk-mixed formulations of identical composition in tumor models remain limited. Microfluidic assembly should therefore be viewed primarily as an enabling strategy for reproducible manufacturing and regulatory compliance rather than a direct enhancer of therapeutic efficacy. The current evidence supports microfluidic assembly as a valuable manufacturing strategy for clinical translation, while acknowledging that formulation composition—polymer chemistry, targeting ligands, cargo selection—likely dominates therapeutic outcome over assembly method per se [70,71]. Table 2 provides a systematic comparison of bulk mixing versus microfluidic assembly across key manufacturing and performance parameters.

### 4.5. Kinetic Versus Thermodynamic Assembly Regimes

Polyplex formation can proceed under kinetic or thermodynamic control, with significant implications for particle stability and long-term behavior. Understanding this distinction informs both formulation design and storage stability assessment. Kinetically trapped structures form when mixing occurs rapidly relative to the timescale of polymer chain reorganization. Under these conditions, initial contact geometries between polymer and nucleic acid become locked into place before equilibrium configurations can be achieved, producing particles that reflect the specific mixing conditions rather than minimum-energy states. Such kinetically trapped polyplexes may be metastable, potentially reorganizing or aggregating over time as chains slowly rearrange toward thermodynamic equilibrium. This behavior has important implications for storage stability and shelf-life assessment [73]. Thermodynamically equilibrated structures, by contrast, form when sufficient time and conditions—elevated temperature, appropriate ionic strength, or extended incubation—permit polymer rearrangement to minimum-energy configurations. These particles are typically more stable over time but may require preparation conditions incompatible with sensitive cargos. mRNA, for example, undergoes rapid hydrolysis at elevated temperatures and cannot tolerate extended incubation periods that might otherwise favor equilibration [73].

Microfluidic assembly occupies an intermediate regime, producing particles under highly reproducible kinetic conditions that nonetheless achieve sufficient local equilibration within the mixing zone for practical stability. The rapid but controlled mixing characteristic of microfluidic devices yields particles that are kinetically reproducible across batches yet sufficiently equilibrated to resist post-formation reorganization. This balance makes microfluidic assembly particularly attractive for formulations requiring both reproducibility and stability [74].

### 4.6. Hierarchy of Complexation Determinants

Integrating the parameters discussed throughout this section, efficient polyplex formation requires optimization across multiple variables that can be conceptually ranked by their relative impact on particle formation and function. The N/P ratio represents the most critical formulation parameter, as it determines whether sufficient cationic charge is present to achieve complete nucleic acid condensation. Below cargo-specific thresholds—typically N/P 3–5 for pDNA, 8–15 for siRNA—complexation remains incomplete regardless of other optimizations. Ionic strength during assembly similarly exerts fundamental influence, as physiological salt concentrations partially screen electrostatic interactions; formulations optimized at low ionic strength may dissociate or aggregate when diluted into biological fluids [12]. Polymer molecular weight must exceed minimum thresholds for effective multivalent binding, below which stable condensation cannot occur regardless of stoichiometry.

Secondary parameters modulate performance within the constraints established by these critical determinants. Polymer architecture and pKa influence both condensation strength and endosomal buffering capacity, determining escape efficiency [62,75]. Assembly method governs reproducibility and size distribution, affecting biodistribution and cellular uptake uniformity. Cargo type dictates specific requirements for N/P ratio, protection stringency, and release kinetics [76].

Fine-tuning parameters—hydrophobic content, temperature, mixing rate, post-formation stabilization—enable optimization once fundamental requirements are satisfied [77]. Hydrophobic modifications can enhance complex stability and membrane interaction; temperature and mixing rate influence the kinetic-thermodynamic balance; post-formation treatments such as lyophilization or PEGylation address storage and circulation stability [75].

For clinical translation, the combination of cargo-appropriate N/P ratio, physiological ionic strength during formulation, and microfluidic assembly with validated process parameters offers the most reproducible path to well-characterized polyplexes meeting regulatory expectations [78]. The hierarchy of determinants provides a rational framework for troubleshooting formulation challenges: if complexation fails, address critical parameters before optimizing secondary or fine-tuning variables [79].

## 5. Mechanisms of Cellular Entry and Intracellular Trafficking

The therapeutic efficacy of polyplexes depends on their ability to navigate a series of biological barriers: cell surface binding, internalization, endosomal escape, cytosolic transport, and—for pDNA—nuclear entry. A central mechanistic question is which of these steps is rate-limiting and how polymer design can address specific bottlenecks. While early studies emphasized cell binding as critical, contemporary evidence identifies endosomal escape as the principal rate-limiting step for most polyplex systems, with only 1–2% of internalized material typically reaching the cytosol [80]. Understanding the interplay between universal trafficking mechanisms and polymer-specific properties is essential for rational carrier design.

### 5.1. Cell Surface Binding and Internalization

Polyplexes engage cells through multiple mechanisms that do not require specific receptor-ligand interactions. Adsorptive endocytosis occurs when cationic polyplex surfaces interact electrostatically with anionic plasma membrane components—heparan sulfate proteoglycans (HSPGs), phospholipids, and sialic acid residues. This non-specific, charge-mediated uptake is the dominant entry pathway for untargeted cationic polyplexes and does not require receptor engagement [81]. HSPGs have been demonstrated to serve as cell-surface receptors for diverse macromolecular cargo including polycation-nucleic acid complexes, with polyplexes captured by syndecan clusters on actin-rich plasma membrane extensions prior to cell entry [82].

Macropinocytosis involves actin-driven membrane ruffling that engulfs extracellular fluid and particles non-selectively. This pathway can internalize polyplexes regardless of surface chemistry and is particularly relevant for larger particles (>200 nm). Macropinocytosis generates large (0.5–5 μm) vesicles that eventually fuse with lysosomes; while non-selective, this pathway can be therapeutically exploited as it is upregulated in many cancer cells [83].

Receptor-mediated endocytosis can be engineered through ligand conjugation to enhance specificity and uptake efficiency. The choice of receptor target should match the intended cell population. For tumor cell targeting, commonly exploited receptors include transferrin receptor (TfR, overexpressed in many cancers), EGFR, folate receptor-α, and integrins (αvβ3, αvβ5) [8]. For cancer vaccine applications targeting antigen-presenting cells (APCs), more relevant receptors include DC-SIGN (dendritic cell-specific), mannose receptor (CD206), CLEC9A (cross-presenting DCs), DEC-205, and Toll-like receptors (TLR3, TLR7/8, TLR9) that can simultaneously provide adjuvant signals. Targeting APC-specific receptors enhances both uptake and immunostimulation—dual functions particularly valuable for vaccine delivery [84].

### 5.2. Endocytic Pathways and Their Implications

Following cell surface engagement, polyplexes are internalized through distinct endocytic routes with different trafficking fates. Clathrin-mediated endocytosis (CME) is the best-characterized pathway, involving clathrin-coated pit formation, dynamin-mediated scission, and delivery to early endosomes (pH ~ 6.5) that mature to late endosomes (pH ~ 5.5) and fuse with lysosomes (pH ~ 4.5). CME is efficient but delivers cargo to degradative compartments, making endosomal escape essential [85].

Caveolae-mediated endocytosis involves flask-shaped membrane invaginations enriched in caveolin-1. This pathway can bypass lysosomal degradation, potentially delivering cargo to the ER or Golgi. However, caveolar uptake is typically slower and lower-capacity than CME. Studies with PEI polyplexes demonstrated that uptake via caveolae resulted in more productive transfection compared to clathrin-mediated uptake. The optimal entry pathway depends on particle properties (size, charge, ligand) and cell type. Importantly, polyplexes often enter via multiple simultaneous pathways, complicating mechanistic analysis. Regardless of entry route, most pathways converge on endosomal compartments requiring escape strategies [86].

### 5.3. Endosomal Escape: The Principal Bottleneck

Following internalization, polyplexes reside in endosomes that progressively acidify and ultimately fuse with lysosomes, where nucleic acids are degraded by nucleases and acidic hydrolases. Escape before lysosomal fusion is typically the rate-limiting step. Quantitative studies using gold-conjugated siRNA and electron microscopy in HeLa cells demonstrated that only 1–2% of internalized LNP-siRNA escapes from endosomes into the cytosol, occurring during a limited time window corresponding to early-to-late endosome transition. Escape efficiency likely varies across cell types, nanoparticle formulations, and cargo characteristics; these values represent a specific LNP system rather than a universal constant for all delivery vehicles [80]. Similar escape efficiencies have been documented for GalNAc-ASO conjugates in hepatocytes in vivo using NanoSIMS microscopy [87].

Several mechanistically distinct escape strategies have been developed:

#### 5.3.1. Proton Sponge Effect

The proton sponge hypothesis proposes that polymers with high buffering capacity in the endosomal pH range (5.0–7.0) resist acidification, causing continued H^+^-ATPase activity with concomitant Cl^−^ and water influx. The resulting osmotic pressure buildup ruptures the endosomal membrane [88].

The physicochemical basis of the proton sponge mechanism rests upon polymers possessing pKa values between 5.5 and 7.0, exemplified by polyethylenimine with its mixture of primary, secondary, and tertiary amines, as well as histidine-containing polymers, which undergo progressive protonation as endosomal pH decreases from physiological 7.4 toward lysosomal 5.0, thereby buffering against compartmental acidification. Each protonated amine necessitates charge-balancing chloride influx, with osmotically obligated water following to maintain ionic equilibrium, and chloride accumulation within endosomes containing PEI has been experimentally confirmed through ion-sensitive fluorescent probes [89,90]. However, while the proton sponge mechanism is widely invoked to explain polyplex-mediated endosomal escape, direct experimental support remains incomplete and somewhat controversial. Benjaminsen and colleagues demonstrated that PEI polyplexes did not significantly alter lysosomal pH compared to untreated controls, challenging the classical interpretation that buffering-induced osmotic swelling drives membrane rupture. Alternative or complementary mechanisms, including direct membrane perturbation by cationic polymer segments interacting with anionic phospholipids, may contribute substantially to escape efficiency. Live-cell imaging studies have visualized burst release of genetic cargo occurring from typically only one or two endosomes per cell, with complete discharge of contents into the cytosol, suggesting localized membrane destabilization events rather than wholesale endosomal lysis [91]. Among commonly employed polymers, branched PEI of 25 kDa exhibits strong buffering capacity owing to its high density of titratable amines, linear PEI demonstrates intermediate buffering, histidinylated polylysine gains pH-responsive buffering through introduction of imidazole side chains, and polyamidoamine dendrimers provide generation-dependent buffering proportional to their surface amine count [89].

#### 5.3.2. Membrane Destabilization and Pore Formation

Direct interactions between cationic polymers and endosomal membranes can induce local destabilization, thinning, or pore formation. Electrostatic disruption occurs when cationic polymer segments interact with anionic phospholipids (phosphatidylserine, phosphatidylglycerol) in the inner endosomal membrane leaflet, inducing lipid reorganization, membrane thinning, and transient pore formation [92].

Hydrophobic insertion represents an additional mechanism: polymers with hydrophobic domains (alkyl chains, aromatic residues) can insert into the lipid bilayer, disrupting membrane integrity. PBAEs with hydrophobic end-caps show enhanced escape through this mechanism. Studies using galectin-8 recruitment assays—which detect endosomal membrane damage through galectin binding to exposed luminal glycans—have demonstrated that PBAE hydrophobicity correlates with endosomal disruption efficiency [35]. Lipophilic PBAEs can destabilize endo-lysosomal membranes through membrane fusion and pore formation mechanisms distinct from the classical proton sponge effect [93].

#### 5.3.3. Safety Considerations for Endosomolytic Agents

Bacterial toxins such as listeriolysin O (LLO) can form membrane pores at endosomal pH. However, LLO is highly immunogenic and potentially cytotoxic, limiting translational potential without substantial protein engineering to reduce immunogenicity while maintaining pore-forming activity. Similar concerns apply to other bacterial-derived pore-forming proteins, and clinical development paths for agents that cause complete endosomal bursting appear limited due to uncontrollable effects on endosomes in many cell types [94].

### 5.4. Cytosolic Trafficking and Cargo Dissociation

Following endosomal escape, polyplexes must dissociate to release functional nucleic acids. Overly stable complexes fail to release cargo; overly weak complexes dissociate prematurely. This balance is cargo-dependent. For siRNA and miRNA, cytosolic delivery is required for incorporation into the RNA-induced silencing complex (RISC). Complete dissociation from the carrier is necessary for RISC loading. Bioreducible polymers exploiting elevated cytosolic glutathione (2–10 mM versus <10 μM extracellular) can trigger selective intracellular release through disulfide bond cleavage [38]. Dual-responsive polyplexes combining pH-sensitivity with redox-responsiveness have demonstrated enhanced siRNA release and gene silencing compared to single-stimulus systems [47].

For mRNA, the cargo must engage ribosomes for translation but benefits from some carrier association to protect against cytosolic RNases during transport to translation machinery. The optimal balance between protection and release differs from siRNA requirements [60]. Regarding intracellular transport, motor proteins (dynein, kinesin) do not bind nucleic acids directly. Rather, they engage adapter proteins or appropriately functionalized carrier components. For directed transport, vectors must incorporate motor-binding sequences or exploit endogenous trafficking pathways [95,96].

### 5.5. Nuclear Entry: The Additional Barrier for pDNA

**Figure 3:**
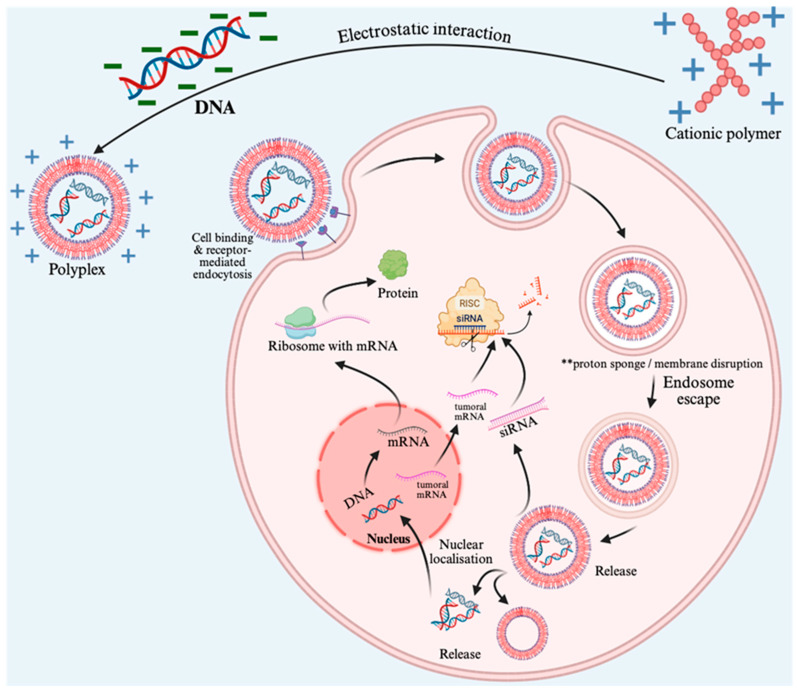
Cationic-polymer polyplex–mediated co-delivery of DNA and siRNA. Plasmid DNA and siRNA form electrostatic polyplexes with cationic polymers, undergo receptor-mediated endocytosis, escape endosomes via proton sponge/membrane disruption, then dissociate to allow nuclear import of DNA for antigen expression and cytosolic loading of siRNA into RISC for tumour mRNA degradation. ** Proton sponge effect: cationic polymer-mediated buffering of endosomal pH causes osmotic swelling and membrane rupture, enabling cytosolic release.

Unlike siRNA and mRNA that function cytoplasmically, plasmid DNA must access the nucleus for transcription, and the nuclear envelope presents a formidable barrier for large complexes. Nuclear pore complexes permit passive diffusion only for molecules below approximately 40 kDa (~9 nm diameter), while larger cargos require active importin-mediated transport recognizing nuclear localization signals [97]. Intact polyplexes typically exceed these size constraints, necessitating substantial dissociation before nuclear entry [98]. Incorporation of nuclear localization signal peptides, particularly the SV40 large T antigen sequence PKKKRKV, enables importin-α/β recognition and active nuclear transport, increasing delivery 10–100-fold in non-dividing cells. In proliferating cells, nuclear envelope breakdown during mitosis provides direct chromatin access without active transport, though polyplexes must survive cytosolic trafficking sufficiently long to exploit this window [99]. Transfection efficiency correlates strongly with proliferation rate, with dividing cells demonstrating 10–100-fold higher expression than quiescent populations—a consideration particularly relevant for cancer applications targeting rapidly dividing malignant cells [99]. Figure 3 provides an integrated schematic of polyplex-mediated co-delivery of DNA and siRNA, illustrating the sequential steps from cellular uptake through endosomal escape to cargo-specific intracellular processing.

## 6. Molecular Therapeutic Strategies with Polyplexes

### 6.1. Plasmid DNA Polyplexes

Plasmid DNA delivery enables expression of therapeutic genes including tumor suppressors, cytotoxic proteins, and immunomodulatory cytokines. Tumor necrosis factor-α (TNF-α) represents an attractive transgene given its potent antitumor activity, though systemic administration causes severe dose-limiting toxicity [100]. Transferrin-targeted PEI polyplexes encapsulating pTNF-α produced sustained intratumoral cytokine expression (48–72 h) in murine models, driving hemorrhagic tumor necrosis and growth inhibition without detectable systemic TNF-α levels [101].

Under optimal in vitro conditions, pDNA polyplexes achieve 10–40% transfection efficiency depending on cell type, polymer composition, and assay methodology; however, in vivo transfection typically drops to <1% due to extracellular barriers, endosomal entrapment, and the additional requirement for nuclear entry. These figures represent ranges across diverse systems rather than universal benchmarks [100]. Unlike siRNA and mRNA that function cytoplasmically, pDNA must access the nucleus for transcription. Nuclear pore complexes permit passive diffusion only for molecules <40 kDa (~9 nm diameter); larger cargos require active importin-mediated transport [102]. Intact polyplexes typically exceed these size constraints, necessitating substantial dissociation before nuclear entry. Incorporation of nuclear localization signal peptides (e.g., SV40 large T antigen sequence PKKKRKV) enables active transport, increasing nuclear delivery 10–100-fold in non-dividing cells. In proliferating cells, nuclear envelope breakdown during mitosis provides direct chromatin access, with transfection efficiency correlating strongly with proliferation rate [103,104].

Key limitations include large cargo size (5–10 kb) resulting in polyplexes of 100–300 nm with limited tumor penetration, reduced efficiency in slowly dividing cells, low but non-zero genomic integration risk and transient expression (days to weeks) [103].

### 6.2. Small Interfering RNA Polyplexes

siRNA polyplexes silence oncogenic transcripts in the cytosol through RNA interference, offering substantial but transient effects. The cyclodextrin-based, transferrin-targeted CALAA-01 represented a landmark as the first targeted siRNA nanoparticle in human cancer trials. This four-component system—cyclodextrin-containing polymer, PEG, human transferrin, and anti-RRM2 siRNA—demonstrated dose-dependent tumor accumulation with qRT-PCR and 5′-RACE confirming RNAi mechanism through detection of predicted mRNA cleavage fragments [105]. However, in 24 patients with solid tumors, no objective responses were observed; the best outcome was stable disease for 4 months in one melanoma patient. The program was discontinued, illustrating the gap between mechanistic proof-of-concept and clinical benefit [106].

siRNA polyplexes achieve 50–90% knockdown in vitro under optimal conditions, but in vivo silencing is typically 30–60% and transient (3–7 days), necessitating repeated administration. Limitations include siRNA instability (serum half-life of minutes without chemical modifications such as 2′-O-methyl or phosphorothioate), off-target silencing through partial sequence complementarity, immune stimulation via TLR3/7/8 and hepatic sequestration diverting material from tumors [107].

### 6.3. MicroRNA Polyplexes

MicroRNA replacement therapy restores tumor-suppressive miRNAs downregulated in cancer. Loss of miR-145 and miR-33a—repressors of c-Myc, ERK5, and Pim-1—is common across cancer types [108]. Systemic PEI polyplex delivery demonstrated target downregulation and tumor inhibition in preclinical models [109]. Delivery of miR-200c inhibited epithelial-to-mesenchymal transition and improved survival in triple-negative breast cancer models [110].

However, PEI biocompatibility remains problematic. Unmodified branched PEI (25 kDa) causes dose-dependent mitochondrial toxicity, membrane disruption, and inflammatory responses. Mechanistic studies demonstrate biphasic cytotoxicity: early membrane damage (<30 min) followed by mitochondrial-mediated apoptosis (~24 h) with impaired electron transport chain function [111]. Successful applications typically require low-MW PEI (≤10 kDa), PEGylation, or biodegradable modifications. miRNA polyplexes achieve 40–70% target repression in vitro, but multitargeting complicates interpretation—effects reflect simultaneous regulation of dozens to hundreds of transcripts [109].

### 6.4. Messenger RNA Polyplexes

mRNA enables transient protein expression without genomic integration and functions in non-dividing cells, but demands stringent RNase protection. PEG-polyaspartate polyplex micelles delivering sFlt-1 mRNA reduced vascular density and suppressed pancreatic tumor growth. mRNA polyplexes achieve 20–60% protein expression in vitro but generally underperform lipid nanoparticles (LNPs), which benefit from optimized ionizable lipid chemistry and extensive clinical validation through COVID-19 vaccines and FDA-approved Onpattro. Polyplexes must demonstrate advantages such as repeated dosing without anti-PEG immunity or superior active targeting to justify development over established LNP platforms [112,113].

### 6.5. CRISPR/Cas Gene Editing

Gene editing expands polyplex applications to permanent genomic changes. Delivery formats include Cas9 pDNA/sgRNA, Cas9 mRNA/sgRNA, or ribonucleoprotein complexes, each with distinct kinetics and safety profiles. Poly(β-amino ester) nanoparticles have enabled in vivo Cas9 mRNA and sgRNA delivery with gene disruption in tumor models [114]. Polyplex-mediated editing achieves 10–40% indel formation in vitro versus 50–80% for optimized LNPs; in vivo rates are generally 5–30%. Limitations include off-target editing concerns, large cargo size (Cas9 pDNA ~ 10 kb), and regulatory complexity for permanent modifications [115].

### 6.6. Short Hairpin RNA Polyplexes

shRNA enables durable gene silencing through continuous expression from DNA templates. The FANG vaccine (Vigil) employs bi-shRNA targeting TGF-β1/β2 combined with GM-CSF, introduced ex vivo into autologous tumor cells. Phase II trials demonstrated immunological responses and survival benefits in selected populations. Limitations include cellular processing saturation (competition with endogenous miRNA for exportin-5 and RISC), immunogenicity from sustained dsRNA expression, and nuclear entry requirements similar to pDNA [116].

## 7. Barriers to Intratumoral Delivery

Effective polyplex-mediated gene therapy requires navigation through a cascade of interconnected biological barriers that function as multiplicative rather than additive obstacles. Following intravenous administration, polyplexes must survive circulation, extravasate across tumor vasculature, penetrate dense extracellular matrix, achieve receptor-mediated cellular uptake, escape endosomal compartments, and release nucleic acid cargo at the appropriate intracellular site of action. Critically, failure at any single step eliminates material from all subsequent stages, while interdependencies between barriers—such as protein corona formation affecting both systemic clearance and ligand accessibility—compound the challenge beyond simple additive effects [117].

### 7.1. Blood Circulation: Protein Corona and Systemic Clearance

Upon entering systemic circulation, polyplexes rapidly adsorb plasma proteins to form a dynamic layer termed the protein corona, which fundamentally redefines their biological identity and fate. This corona exhibits a hierarchical structure comprising two distinct layers: an inner hard corona of tightly bound proteins—including albumin, immunoglobulins, complement components, and apolipoproteins—characterized by slow exchange kinetics over hours to days, and an outer soft corona of loosely associated proteins undergoing rapid dynamic exchange within seconds to minutes. The hard corona composition, rather than the engineered particle surface, ultimately determines cellular interactions, biodistribution patterns, and clearance kinetics [118].

Corona formation has profound implications for actively targeted polyplexes. Surface-conjugated targeting ligands can become buried within the protein corona, rendering them sterically inaccessible for receptor engagement—a phenomenon termed corona masking. This effect may explain why many actively targeted nanoparticles demonstrate only marginal improvement over untargeted controls in vivo despite excellent in vitro receptor specificity. Strategies to address corona masking include presentation of targeting ligands on extended polyethylene glycol chains that protrude beyond the corona layer, corona-resistant surface chemistries such as zwitterionic or polysarcosine coatings, and pre-formed biomimetic coronas designed to minimize subsequent protein adsorption [119].

Corona proteins trigger recognition and clearance by the mononuclear phagocyte system through multiple receptor-mediated mechanisms: Fc receptors binding adsorbed immunoglobulins, scavenger receptors (SR-A, MARCO) recognizing denatured or misfolded proteins, and complement receptors engaging opsonins such as C3b and iC3b. Kupffer cells residing in hepatic sinusoids and splenic macrophages constitute the primary clearance sites, typically sequestering greater than 80% of administered nanoparticle doses [120]. A landmark meta-analysis examining nanoparticle delivery across 117 preclinical studies revealed that only 0.7% median of administered dose reaches solid tumors in mouse models, with even lower accumulation expected in human cancers exhibiting less pronounced enhanced permeability and retention effects. This figure encompasses substantial heterogeneity across nanoparticle types, tumor models, and measurement methodologies; accumulation in human cancers—characterized by slower growth rates and more mature vasculature—is generally expected to be lower, though direct comparative data remain limited [121].

Polyethylene glycol conjugation remains the most widely employed strategy for reducing corona formation and extending circulation half-life, yet PEG presents significant limitations that increasingly constrain its utility. The PEG dilemma refers to the fundamental tradeoff wherein PEG reduces protein binding and phagocytic clearance but simultaneously impairs cellular uptake and endosomal escape, potentially compromising transfection efficiency. More concerning, pre-existing anti-PEG antibodies occur in 25–72% of treatment-naïve populations depending on detection methodology, antibody isotype measured, and geographic population studied, due to widespread PEG exposure through cosmetics, foods, and pharmaceutical excipients. These antibodies can trigger immediate hypersensitivity reactions upon first exposure to PEGylated therapeutics, while repeated administration induces anti-PEG IgM production leading to accelerated blood clearance of subsequent doses and potentially complete therapeutic failure [122].

### 7.2. Hemotoxicity and Complement Activation

Cationic polyplexes present specific hematological risks beyond mononuclear phagocyte system clearance [123]. Strongly cationic surfaces can trigger complement cascades through both the classical pathway via antibody-mediated activation and the alternative pathway through spontaneous C3 hydrolysis on particle surfaces. Complement activation generates the anaphylatoxins C3a and C5a, potent inflammatory mediators causing vasodilation, hypotension, bronchospasm, and cutaneous reactions—collectively termed complement activation-related pseudoallergy (CARPA). Clinical manifestations range from mild infusion reactions manageable with premedication to life-threatening anaphylactoid responses requiring immediate intervention [124].

Cationic polymers interact with negatively charged erythrocyte membranes, inducing dose-dependent hemolysis, aggregation, and morphological alterations including echinocyte formation. Polyethylenimine and polyamidoamine dendrimers demonstrate hemolytic activity correlating directly with surface amine density and zeta potential [125]. Additionally, cationic nanoparticles activate platelets through charge-mediated interactions, promoting aggregation and potentially contributing to thrombotic complications. Blood cell aggregation in pulmonary capillaries following intravenous injection of highly cationic polyplexes has been documented, causing acute respiratory distress that can become dose-limiting in clinical applications [126].

### 7.3. Vascular Barrier

Macromolecular transport across tumor endothelium relies upon the enhanced permeability and retention (EPR) effect—the pathophysiological phenomenon of leaky vasculature and impaired lymphatic drainage characteristic of tumor angiogenesis [127]. Tumor vessels exhibit enlarged interendothelial junctions with fenestrations ranging from 100 to 600 nm and discontinuous basement membranes, theoretically permitting extravasation of appropriately sized nanoparticles. However, EPR demonstrates marked heterogeneity across tumor types, anatomical locations, and individual patients, and appears substantially less pronounced in human cancers than in rapidly growing murine xenografts [128].

Importantly, contemporary evidence challenges the passive EPR paradigm. Sindhwani and colleagues demonstrated that approximately 97% of nanoparticle tumor entry occurs through active transcytosis rather than passive paracellular transport through endothelial gaps in subcutaneous tumor xenograft models using gold and silica nanoparticles (50–100 nm); whether this mechanism predominates across different particle compositions, sizes, and human tumor types requires further investigation [128]. Factors substantially reducing EPR-mediated accumulation include slow-growing well-differentiated tumors with mature vasculature, hypovascular tumor types such as pancreatic adenocarcinoma, central tumor regions with mechanically compressed vessels, and prior antiangiogenic therapy that normalizes vascular architecture [129].

### 7.4. Tumor Interstitium and Stromal Barriers

Following extravasation, polyplexes must navigate the tumor interstitium—a dense, heterogeneous microenvironment fundamentally distinct from normal tissue architecture [121]. Dense stromal networks comprising collagen types I, III, and IV, hyaluronan, proteoglycans, and elastin create formidable physical barriers impeding particle diffusion. Effective diffusivity decreases exponentially with increasing particle size, with particles exceeding 60 nm experiencing severe restriction in collagen-rich stromal regions [130]. The extracellular matrix carries net negative charge, creating electrostatic repulsion for anionic nanoparticles while potentially trapping cationic polyplexes through nonspecific binding interactions [121].

Elevated interstitial fluid pressure represents an additional transport barrier arising from rapid tumor proliferation, vascular hyperpermeability, and dysfunctional lymphatic drainage. Interstitial pressure in tumor cores can approach or exceed intravascular pressure, eliminating the convective transport gradient that normally facilitates drug penetration and potentially driving fluid backflow that actively expels nanoparticles from tumor parenchyma [127].

Tumor-associated macrophages, frequently comprising 30–50% of tumor mass in certain cancer types, represent a major sink diverting nanoparticle accumulation away from malignant cells. Macrophage uptake is enhanced by several polyplex properties: cationic surface charge promotes scavenger receptor engagement, larger particles exceeding 100 nm favor phagocytic uptake over passive diffusion to cancer cells, protein corona opsonization provides Fc receptor ligands, and phosphatidylserine exposure on apoptotic-mimetic surfaces triggers efferocytic responses. Whether macrophage sequestration is problematic—diverting therapeutic payload from target cancer cells—or potentially beneficial through enabling macrophage reprogramming or sustained local release depends upon the specific therapeutic strategy employed [128].

### 7.5. Strategies to Overcome Delivery Barriers

#### 7.5.1. Size-Switching Nanoparticles

Size-switching systems maintain larger dimensions of 80–150 nm during systemic circulation for favorable pharmacokinetics and reduced renal clearance, then undergo triggered reduction to 20–40 nm within the tumor microenvironment to enhance interstitial penetration. Environmental triggers exploited for size transition include pH-responsive mechanisms utilizing acid-labile bonds such as hydrazones, acetals, and ortho esters that exploit tumor acidosis at pH 6.5–6.8; enzyme-responsive systems incorporating matrix metalloproteinase or cathepsin-cleavable linkers that respond to elevated protease activity in tumor stroma; and externally applied triggers including light, ultrasound, or magnetic fields enabling spatial and temporal control albeit requiring interventional procedures [131].

#### 7.5.2. Charge-Reversal Systems

Charge-reversal polyplexes present neutral or anionic surfaces during circulation to minimize corona formation, complement activation, and nonspecific vascular binding, then transition to cationic character within the tumor microenvironment or endosomal compartments to facilitate cellular uptake and membrane disruption for endosomal escape [132]. Common mechanisms include pH-responsive charge conversion through cleavable amide bonds linking anionic shielding groups such as citraconic or dimethylmaleic anhydride that hydrolyze under mildly acidic conditions to expose underlying amine functionalities; enzyme-cleavable shielding with anionic polymeric coatings connected via protease-sensitive linkers; and redox-responsive designs featuring disulfide-linked anionic domains that detach upon exposure to elevated glutathione concentrations in the reducing tumor microenvironment or cytosol [133].

#### 7.5.3. Tumor-Penetrating Peptides: iRGD Versus Classical RGD

Tumor-penetrating peptides enhance intratumoral distribution through distinct mechanisms requiring clear differentiation. Classical RGD peptides in linear or cyclic configurations bind αvβ3 and αvβ5 integrins expressed on tumor vasculature and some cancer cells, providing vascular targeting and enhanced endothelial adhesion. However, RGD-targeted nanoparticles largely remain confined to perivascular regions without achieving deep tissue penetration into avascular tumor cores [134].

The internalizing RGD peptide iRGD (sequence CRGDK/RGPD/EC) functions through a distinct multi-step mechanism enabling substantially broader distribution. Initial binding to αvβ3/αvβ5 integrins on tumor endothelium positions the peptide for proteolytic cleavage by tumor-associated proteases, exposing a cryptic C-end Rule (CendR) motif with the consensus sequence R/KXXR/K. This activated CendR motif binds neuropilin-1 (NRP-1), triggering a transcytosis and tissue-penetration pathway that transports cargo deep into extravascular tumor parenchyma. Remarkably, co-administered free iRGD can enhance tumor penetration of separately injected therapeutics through this bystander effect, eliminating the requirement for covalent conjugation [135].

#### 7.5.4. Extracellular Matrix Modification

Rather than engineering nanoparticles to navigate existing stromal barriers, therapeutic strategies can actively modify the extracellular matrix to enhance penetration. Hyaluronidase co-delivery enzymatically degrades hyaluronan, reducing interstitial viscosity and normalizing elevated fluid pressure. Collagenase administration or lysyl oxidase inhibition targets collagen barriers, although concerns regarding potential metastasis promotion require careful consideration. Angiotensin receptor blockers such as losartan reduce stromal collagen and hyaluronan deposition through transforming growth factor-β signaling inhibition, representing a clinically approved approach for stromal normalization [127].

## 8. Safety Considerations and Toxicities

Safety remains a critical barrier limiting clinical translation of polyplex-based therapeutics. Despite advances in nanoparticle engineering improving delivery efficiency, systemic administration of cationic carriers continues to elicit toxicities constraining maximum tolerated doses and therapeutic indices. Understanding the mechanistic basis of toxicity and its dependence upon polymer structure is essential for rational carrier design [123].

### 8.1. The Central Role of Cationic Charge in Toxicity

The cationic charge essential for efficient nucleic acid condensation is simultaneously the primary driver of polyplex toxicity. Positively charged polymers interact electrostatically with negatively charged biological structures throughout the body, producing toxicity through multiple mechanisms [123].

Plasma membrane disruption occurs through cationic polymer binding to anionic membrane components including phosphatidylserine, phosphatidylglycerol, and heparan sulfate proteoglycans, inducing membrane thinning, nanoscale pore formation, and phospholipid extraction. These perturbations trigger immediate necrotic cell death characterized by lactate dehydrogenase release and uncontrolled calcium influx [123]. Cationic polymers also interact with the negatively charged erythrocyte glycocalyx, causing dose-dependent hemolysis, cell aggregation, and morphological transformation to echinocyte forms. Polyethylenimine and polyamidoamine dendrimers demonstrate hemolytic activity correlating directly with surface amine density and measured zeta potential [125].

Cationic polyplexes bind vascular endothelium, inducing membrane disruption, increased paracellular permeability, and inflammatory endothelial activation contributing to vascular leak syndrome observed following high-dose administration. Platelet activation through charge-mediated surface interactions promotes aggregation and potentially contributes to thrombotic complications, particularly concerning in patients with pre-existing cardiovascular disease [126]. As detailed in Section 7.2, strongly cationic surfaces trigger complement cascades through alternative pathway activation, generating anaphylatoxins responsible for complement activation-related pseudoallergy ranging from mild infusion reactions to potentially fatal anaphylactoid responses [124].

### 8.2. Structural Determinants of Polymer Toxicity

Cationic polymer toxicity is governed by molecular weight, architecture, and biodegradability, each presenting distinct optimization trade-offs (Table 3).

These structural parameters create a fundamental optimization paradox: lower molecular weight and linear architectures reduce toxicity but diminish nucleic acid condensation and endosomal escape efficiency. Biodegradable designs decouple extracellular stability from intracellular degradation, enabling high-MW function without long-term accumulation [123].

### 8.3. PEI Toxicity: Detailed Mechanistic Analysis

Polyethylenimine remains the most extensively studied polycationic gene carrier and illustrates key toxicity mechanisms relevant across polymer classes. PEI cytotoxicity unfolds through two kinetically and mechanistically distinct phases [52].

The early phase occurring within 30 min involves PEI binding to plasma membrane proteoglycans including syndecans and glypicans, direct membrane disruption evidenced by lactate dehydrogenase release, calcium influx triggering immediate necrotic features, and membrane pore formation visualized by electron microscopy. The late phase manifesting around 24 h involves mitochondrial accumulation and membrane potential depolarization, cytochrome c release through nanoscale pore formation in mitochondrial membranes, activation of caspase-3 and caspase-9 initiating the intrinsic apoptotic pathway, and ATP depletion producing bioenergetic crisis [52]. The approximately 10-fold toxicity differential between 25 kDa and 2 kDa PEI provides a practical design principle: employ the lowest molecular weight compatible with effective condensation, or utilize biodegradable crosslinking strategies enabling high molecular weight function with subsequent degradation [138].

### 8.4. Immunogenicity and Repeat-Dose Considerations

Beyond acute toxicity, immunological responses substantially limit repeated polyplex administration required for cancer therapy. Pre-existing anti-PEG antibodies with prevalence ranging from 25 to 72% across studied populations can trigger immediate hypersensitivity upon initial exposure to PEGylated formulations, while repeated administration induces anti-PEG IgM production causing accelerated blood clearance of subsequent doses. This accelerated clearance phenomenon can reduce circulation half-life by greater than 90%, potentially eliminating therapeutic efficacy in multi-cycle treatment regimens [122].

Antibodies directed against polymer backbones beyond PEG—including responses to chitosan, polyamidoamine, and modified polyethylenimine—can develop following repeated exposure, though these remain less well characterized than anti-PEG responses. Cationic polymers can also function as immune adjuvants, potentially beneficial for vaccine applications but problematic for therapeutic gene delivery requiring repeated dosing without inflammatory stimulation [52,123,139].

## 9. Clinical Development Status

Despite decades of preclinical development demonstrating promising efficacy in laboratory models, clinical translation of polyplex-based therapeutics for oncology applications has proven markedly limited. No polyplex formulation has achieved regulatory approval for cancer indications to date. This section provides critical evaluation of accumulated clinical experience, identifies recurring developmental challenges, and assesses realistic prospects for future advancement.

### 9.1. CALAA-01: The Most Advanced Polyplex Program

CALAA-01, a transferrin receptor-targeted cyclodextrin-polymer nanoparticle delivering siRNA against ribonucleotide reductase M2 subunit (RRM2), represents the most extensively characterized polyplex system evaluated in human cancer trials. This four-component formulation comprising cyclodextrin-containing polymer, adamantane-polyethylene glycol stabilizer, transferrin targeting ligand, and anti-RRM2 siRNA underwent Phase Ia/Ib evaluation in 24 patients with refractory solid tumors [106].

The study demonstrated dose-dependent pharmacokinetics consistent with preclinical predictions and, critically, tumor biopsy analysis confirmed nanoparticle localization within malignant tissue. Most significantly, 5′-RACE analysis detected RRM2 mRNA cleavage products at the predicted siRNA-mediated site in patient tumor samples—providing first-in-human evidence that systemically administered siRNA could engage the RNA interference machinery within solid tumors [105,106].

Despite this mechanistic validation, clinical outcomes proved disappointing. No objective tumor responses were observed across the entire study population; best clinical response was stable disease. Dose-limiting toxicities emerging at higher dose levels included fatigue, hypersensitivity reactions, and ischemic colitis. Transient dose-dependent thrombocytopenia occurred across multiple dose cohorts. Development was subsequently discontinued despite proof-of-mechanism, reflecting the gap between demonstrating target engagement and achieving clinical benefit [105,106].

Critical interpretation reveals that CALAA-01 successfully demonstrated polyplex-mediated siRNA can reach human tumors and engage intended molecular targets following systemic administration. However, target engagement alone proved insufficient for therapeutic effect. Several hypotheses explain this disconnection: RRM2 knockdown as single-agent therapy may be insufficient against treatment-refractory disease; achieved knockdown magnitude, while detectable, may have remained below therapeutic thresholds; and patient population selection without predictive biomarkers may have included tumors inherently resistant to this therapeutic approach [105,106].

### 9.2. EGEN-001: Intraperitoneal IL-12 Plasmid Delivery

EGEN-001, comprising polyethylenimine-polyethylene glycol-cholesterol lipopolymer complexed with interleukin-12 encoding plasmid DNA, underwent Phase II evaluation in 20 patients with platinum-resistant ovarian cancer through intraperitoneal administration. No objective tumor responses were observed; 35% of patients achieved stable disease with 30% demonstrating progression-free survival exceeding 6 months. Median progression-free survival reached only 2.9 months with median overall survival of 9.2 months. Common adverse events including fatigue, fever, chills, and abdominal discomfort remained grade 1–2 in severity, though three patients withdrew due to tolerability concerns. Development was not pursued beyond this trial [140].

The absence of objective responses in this heavily pretreated population suggests several potential limitations: insufficient IL-12 expression levels achieved through plasmid transfection, inadequate penetration of intraperitoneally administered polyplexes into tumor nodules, or intrinsic limitations of immunotherapy approaches in platinum-resistant disease characterized by immunosuppressive tumor microenvironments [140]. Table 4 summarizes key preclinical studies demonstrating polyplex-mediated gene therapy across diverse tumor types and nucleic acid cargos. Table 5 presents the clini-cal trials conducted to date with polyplex and lipoplex-based cancer gene therapeutics, highlighting the gap between preclinical promise and clinical outcomes.

These data illustrate both the breadth of preclinical polyplex applications—spanning siRNA, miRNA, mRNA, pDNA, and CRISPR across diverse tumor types—and the translational challenges evident in clinical trials. While preclinical studies consistently demonstrate target engagement and antitumor effects, clinical translation has yielded mechanistic proof-of-concept (CALAA-01) but limited objective responses. The most encouraging clinical signals (SGT-94) employed receptor-targeted delivery with confirmed tumor-selective gene expression, suggesting that active targeting and patient selection may be critical for future success.

## 10. Discussions

Polyplexes have evolved from simple electrostatic condensates into sophisticated multi-stage delivery vehicles incorporating stimulus-responsive elements, active targeting moieties, and engineered trafficking sequences. This review has examined the current state of the field with emphasis on both demonstrated capabilities and persistent limitations. Critical assessment of accumulated evidence reveals a platform with confirmed mechanistic validity but substantial translational challenges demanding honest acknowledgment rather than continued optimism.

Clinical translation of polyplex-based cancer therapeutics has proven disappointing despite three decades of preclinical investigation. The extensive preclinical literature contrasts starkly with limited clinical success [123]. In vitro studies routinely achieve 50–90% target knockdown or gene expression, results often not replicated in vivo [52]. Mouse xenograft models frequently demonstrate 40–80% tumor growth inhibition, yet these overestimate human tumor accessibility due to exaggerated enhanced permeability and retention effects in rapidly growing subcutaneous implants [128]. Phase I trials have confirmed delivery mechanisms but target engagement has not translated to objective tumor responses [106]. Phase II evaluation has yielded limited or no objective responses with no polyplex products advancing toward regulatory approval [140]. Multiple factors contribute to this translational gap. Biological factors include xenograft models overestimating EPR effect and tumor accessibility compared to human cancers; greater heterogeneity in receptor expression, stromal architecture, and immune contexture across human tumors; and immunocompetent model limitations failing to predict immunogenicity and repeat-dose tolerability [128]. Technical factors include inconsistent physicochemical characterization across preclinical studies, absence of standardized reporting for critical parameters, and batch-to-batch variability in polymer synthesis and polyplex assembly [123]. Clinical development factors include enrollment of unselected patient populations without biomarker guidance, single-agent studies against targets already addressed by validated small molecule or antibody therapies, and insufficient dose optimization constrained by early toxicity emergence [106].

It is important to distinguish between clinically validated advantages—demonstrated through regulatory approval and large-scale human use—and theoretical or preclinical advantages that remain to be confirmed in comparative clinical settings. Lipid nanoparticles have achieved regulatory approval for siRNA therapeutics (patisiran/Onpattro for hereditary transthyretin amyloidosis) and demonstrated massive clinical validation through mRNA vaccines administered to billions of individuals during the COVID-19 pandemic [160]. Multiple lipid nanoparticle formulations have progressed to Phase III evaluation for various indications, manufacturing at commercial scale is well-established, and substantial pharmaceutical industry investment has driven continuous optimization [112].

Polyplexes have not received comparable investment or systematic optimization effort, rendering direct efficacy comparisons premature and potentially misleading. Lipid nanoparticle success was propelled by intensive medicinal chemistry optimization of ionizable lipid components, sustained investment from major pharmaceutical companies, fortuitous timing with urgent pandemic vaccine development needs, and established manufacturing infrastructure enabling rapid scale-up. Whether polyplexes offer sufficient advantages to justify continued development investment in competition with this established platform remains an open question requiring honest assessment [123].

Productive advancement requires several strategic reorientations. First, explicit acknowledgment of current limitations would benefit the field more than continued optimism unsupported by clinical data. Assertions that polyplexes are positioned for imminent clinical translation or that clear trajectories toward approval exist are not substantiated by available evidence. Progress requires identifying and addressing specific failure modes rather than assuming incremental engineering optimization will overcome fundamental biological barriers.

Second, development should focus on contexts where polyplex properties confer genuine comparative advantages rather than competing directly with lipid nanoparticles across all nucleic acid delivery applications. Potential niches include locoregional delivery routes—intratumoral, intraperitoneal, intrathecal administration—bypassing systemic circulation barriers; repeated dosing applications where anti-PEG immunity progressively limits lipid nanoparticle performance; targeting strategies requiring high ligand density achievable through polymer functionalization; and large cargo delivery such as CRISPR components where lipid nanoparticle capacity limitations emerge.

Third, rigorous translational standards must be implemented including standardized reporting of physicochemical parameters at physiological ionic strength rather than deionized water, manufacturing process controls compatible with good manufacturing practice requirements, systematic immunogenicity assessment including anti-polymer antibody monitoring and complement activation markers, and chronic accumulation studies for non-biodegradable polymer systems. Fourth, biomarker-guided development strategies should incorporate patient selection based on confirmed receptor expression, imaging-based validation of tumor accumulation prior to efficacy assessment, and pharmacodynamic markers confirming adequate target engagement.

## 11. Conclusions

Polyplexes represent a modular and chemically versatile platform with demonstrated capacity for targeted nucleic acid delivery and unequivocal proof-of-mechanism in human tumors. However, the persistent gap between preclinical promise and clinical outcomes—characterized by absence of objective tumor responses in completed trials and no products approaching regulatory approval—demands honest reassessment rather than continued optimism.

The evidence reviewed supports several conclusions: polyplexes can deliver nucleic acids to human tumors following systemic administration, but delivery efficiency remains below therapeutic thresholds for most applications; safety concerns arising from cationic charge-mediated toxicity and polymer immunogenicity constrain dosing and therapeutic indices; competition from lipid nanoparticles—which have received substantially greater investment and achieved clinical validation—challenges commercial rationale for polyplex development absent clear differentiating advantages; and specific applications exploiting unique polyplex properties may represent realistic niches where platform characteristics provide genuine benefit over established alternatives.

Productive advancement requires scientific rigor in characterization and reporting, commercial realism regarding competitive positioning, and honest acknowledgment of both achievements and limitations. The opportunity lies not in pursuit of a single breakthrough formulation but in disciplined platform development matching polymer properties to specific clinical contexts where comparative advantages exist and can be validated. Polyplexes are unlikely to supplant lipid nanoparticles as general-purpose nucleic acid delivery vehicles. Their future—if one exists in clinical oncology—resides in applications where unique properties provide clear, demonstrated advantages over established alternatives.

## Figures and Tables

**Table 1:** Values at physiological ionic strength (150 mM NaCl, pH 7.4). Zeta potentials > +20 mV have been associated with increased cytotoxicity in multiple in vitro cell line studies, though the threshold varies with polymer chemistry, cell type, and exposure duration [19]. These values should be considered indicative rather than absolute cutoffs.

Polymer	Size (nm)	ζ-Potential (mV)	Endosomal Escape	Biodegradable	Toxicity	Key Limitations
PEI (branched, 25 kDa)	50–200	+25 to +40	Excellent	No	High	Mitochondrial toxicity; accumulation
PLL	80–300	+20 to +35	Poor	Yes	Moderate	Requires endosomolytic agents
PAMAM (G5–G7)	100–250	+15 to +30	Good	Moderate	Moderate-High	Cost; generation-dependent toxicity
PBAE	100–300	+10 to +25	Good	Excellent	Low	Batch variability; storage instability
Chitosan	150–400	+5 to +20	Moderate	Excellent	Low	pH-dependent solubility
Sequence-defined	50–150	Tunable	Tunable	Tunable	Low	Synthetic complexity; cost

**Table 2:** Comparison of Bulk Mixing versus Microfluidic Assembly for Polyplex Production [54,70,71,72].

Parameter	Bulk Mixing	Microfluidic Assembly
Operational complexity	Simple; minimal equipment	Requires specialized devices and pumps
Polydispersity index (PDI)	0.2–0.4 (typical)	<0.1 (optimized systems)
Batch reproducibility	Variable; operator-dependent	High; defined by device geometry
Size tunability	Limited; primarily via formulation	Adjustable via flow rate ratios
Scalability	Direct scale-up challenging	Parallelization strategies available
GMP compatibility	Requires extensive validation	Compatible with PAT and continuous manufacturing
Cargo compatibility	Suitable for all nucleic acids	May require optimization for sensitive cargos
Evidence for superior efficacy	Established preclinical track record	Manufacturing advantages clear; efficacy advantage unproven

**Table 3:** Structural Determinants of Cationic Polymer Toxicity.

Parameter	Toxicity Mechanism	Quantitative Data	Mitigation Strategy	Reference
Molecular Weight	Higher MW → stronger multivalent membrane binding, greater mitochondrial accumulation, reduced renal clearance, prolonged tissue exposure	IC50: 25 kDa PEI = 5–20 μg/mL vs. 2 kDa PEI = 50–200 μg/mL (~10-fold difference)	Crosslink low-MW polymers via biodegradable bonds; use high-MW at reduced doses with targeting ligands	[123,136]
Architecture	Branched > linear toxicity at equivalent MW; higher local charge density at branch points; greater primary amine proportion; globular conformation concentrates surface charge	PAMAM dendrimers: G5 > G4 > G3 toxicity, correlating with surface amine count	Partial acetylation or hydroxylation of surface amines; linear polymer variants	[137,138]
Biodegradability	Non-degradable polymers (PEI, high-gen PAMAM) resist enzymatic degradation; accumulate in liver, kidney, spleen over weeks–months; trigger chronic inflammation and fibrosis	Fluorescent tracking: PEI persistence for weeks–months post-administration	Cleavable linkages: ester (PBAEs), disulfide (bioreducible), acetal/ketal (pH-sensitive), peptide bonds	[139,140]

**Table 4:** Preclinical Studies of Polyplex-Mediated Cancer Gene Therapy.

Polyplex System	Cargo	Cancer Type	Key Findings	Reference
PEG-PAsp(DET) block/homo polyplex micelles	sFlt-1 mRNA	Pancreatic adenocarcinoma	Enhanced transfection with H integration; significant tumor suppression via anti-angiogenic effect	[141]
Block/homo polyplex micelles	SART3 + CD40L + GM-CSF pDNA	Solid tumors (peritoneal/s.c.)	DC activation; CD4+/CD8+ T cell infiltration; T cell-dependent antitumor efficacy	[142]
Mixed polyplex micelles	SART3 + CD40L pDNA	CT26 colon carcinoma	Subcutaneous tumor size reduction via DNA vaccine approach	[143]
ELR-MUC1 aptamer polyplexes	pDNA	Breast cancer	Self-assembling; MUC1-targeted; selective tumor cell delivery	[144]
PLL-PEG/Chol-DsiRNA	STAT3 siRNA	Breast cancer	Therapeutic activity in early-stage tumors; Phase I candidate	[145]
hPPCs/PBAE NPs	CRISPR/Cas9 (HPV E7)	Cervical cancer (HPV+)	E7 oncogene cleavage; tumor inhibition in SiHa/HeLa cells and xenografts	[146]
PBAE polyplexes	CpG ODN	Melanoma	Dose-dependent DC uptake; improved survival; reduced tumor burden (N:P 1:5 optimal)	[147]
PEG/FA-PEI-PCL	miR-210	Breast cancer (MDA-MB-231)	FA-targeted; 3-layer structure; tumor growth inhibition; enhanced miR-210 expression	[3]
mPEG-pDMAEMA	pDNA (~162 nm)/siRNA (~25 nm)	Ovarian cancer spheroids	siRNA polyplexes penetrated deeper than pDNA; ~50% gene silencing; size-dependent distribution	[18]
gp100-RRRR/CpG-ODN polyplex	Peptide vaccine + adjuvant	Melanoma	Iontophoretic delivery; tumor regression; IFN-γ↑; CD8+ infiltration; prophylactic + therapeutic	[148]
PEI-GA-Lau7	Survivin siRNA	Breast cancer (MDA-MB-231)	~95% uptake; >90% gene silencing; up to 98% cell death	[149]
PEI-PEG-GE11	polyIC	EGFR+ tumors	EGFR-targeted without receptor activation; submicromolar affinity; potent antitumor effects	[150]
Hypericin lipopolyplexes	pDNA (luciferase)	Hepatocellular carcinoma	Light-triggered (587 nm); 60–75× transfection increase; PCI-enhanced endosomal escape	[151]
CCK-BR-targeted PEG-PLL	KRAS G12D siRNA	Pancreatic cancer	Halted PanIN progression; reduced fibrosis/M2 macrophages; improved survival; no off-target toxicity	[152]
PCX-cholesterol polyplexes	NCOA3 siRNA + CXCR4 antagonist	Pancreatic cancer	Dual function; NCOA3 silencing + CXCR4 blockade; reduced metastasis; improved perfusion	[153]
H3K(+H)4b HK peptide	Raf-1 siRNA	MDA-MB-435 xenografts	~50% tumor reduction (3× IV); decreased Raf-1; increased apoptosis; minimal normal tissue toxicity	[154]
F68-PEI polyplexes	pDNA	Oral cancer (SCC-9)	Pluronic-modified; <200 nm with F68 doping; outperformed 25 kDa PEI; best for local delivery	[155]
TMAB-chitosan	pDNA	HEK293T/K562	30–35% transfection (HEK293T); low cytotoxicity; potential for ex vivo applications	[156]
PEI-microbubble hybrids	pDNA (luciferase)	SKNEP-1 kidney tumors	Ultrasound-triggered; >10× in vivo bioluminescence; >40× ex vivo luciferase vs. controls	[157]

**Table 5:** Clinical Trials of Polyplex and Lipoplex-Based Cancer Gene Therapeutics.

Agent	Phase	Cargo/Target	Cancer Type	N	Key Outcomes	Reference
CALAA-01	Ia/Ib	siRNA targeting RRM2; Tf-targeted cyclodextrin polymer	Solid tumors (melanoma)	24	RNAi mechanism confirmed (mRNA cleavage); dose-dependent tumor accumulation; no objective responses; DLTs at higher doses (fatigue, hypersensitivity)	[106]
EGEN-001	II	IL-12 pDNA; PEG-PEI-cholesterol lipopolymer	Platinum-resistant ovarian cancer	20	IP delivery; 0% objective response; 35% stable disease; median PFS 2.9 mo, OS 9.2 mo; manageable toxicity (fatigue, fever, chills)	[140]
SGT-94	I	RB94 pDNA; anti-TfR scFv-targeted liposome	Metastatic genitourinary cancers	11	1 CR (durable), 1 PR, SD; tumor-selective gene delivery confirmed; well tolerated; no DLTs up to 2.4 mg DNA	[158]
DOTAP:Chol-TUSC2	I	TUSC2 (FUS1) pDNA; DOTAP:cholesterol lipoplex	Metastatic lung cancer	31	MTD 0.06 mg/kg; gene transfer confirmed (7/8 biopsies); 5 SD (2.6–10.8 mo); 2 minor regressions; DLTs: hypophosphatemia	[159]

## Data Availability

No new data were created or analyzed in this study. Data sharing is not applicable.
